# SAS-PRP Study: A Real-Life Satisfaction Assessment in Patients with Cartilage Lesions of the Knee Treated by Platelet-Rich Plasma

**DOI:** 10.3390/bioengineering10111276

**Published:** 2023-11-02

**Authors:** Romain Verron, Lucie Zhang, Hélène Bisseriex, Ronan Grimandi, Alix Verrando, Claire Verdaguer, Marie Thomas, Julia Facione, Leo Borrini

**Affiliations:** 1 Physical and Rehabilitation Department, HIA Percy, 92140 Clamart, France; alix.verrando@intradef.gouv.fr (A.V.); claire.verdaguer@intradef.gouv.fr (C.V.); marie.thomas@intradef.gouv.fr (M.T.); julia.facione@intradef.gouv.fr (J.F.); leo.borrini@intradef.gouv.fr (L.B.); 2 Pneumology Department, HIA Clermont-Tonnerre, 29200 Brest, France; lucie.zhang@intradef.gouv.fr; 3 Physical and Rehabilitation Department, HIA Clermont-Tonnerre, 29200 Brest, Franceronan.grimandi@intradef.gouv.fr (R.G.); 4 ORPHY, EA4324, University of Brest, 29238 Brest, France

**Keywords:** platelet-rich plasma, osteoarthritis, knee, cartilage, intra-articular injection, SATMED-Q©

## Abstract

Platelet-rich plasma (PRP) is a rising therapy treating locomotor system lesions such as knee osteoarthritis. The objective of this study was to evaluate patients’ satisfaction 6 to 12 months after a PRP injection for cartilage lesions of their knee under real-life conditions. Patients’ satisfaction was assessed by a specific questionnaire named SATMED-Q©, which explores six different dimensions of a given treatment. In addition, pain and function were assessed thanks to VAS pain, WOMAC, and IKDC scores. Responders were identified through the OMERACT-OARSI criteria. We observed excellent satisfaction after a PRP injection with a SATMED-Q© score of 80.81% 6 to 12 months after the procedure. Even when there was no significant improvement in pain and function scores, 52% of the evaluated population fulfilled the OMERACT-OARSI criteria and were considered responders. According to the sub-group analysis, patients with less osteoarthritis damage (i.e., Kellgren–Lawrence grades 1–2) and older study subjects (i.e., >40 years old) with focal chondropathy had benefited most from their PRP injection. Thus, platelet-rich plasma seems to be a well-tolerated and efficient therapy for cartilage lesions of the knee.

## 1. Introduction

The latest discoveries concerning platelet-rich plasma (PRP) revealed its consequent complexity in its composition and mechanisms of action. Probably underestimated or entirely unknown in early stages [1,2,3], the large amount of PRP components creates a real environment of bioactive factors [4]. Simultaneously bringing platelets’ catabolic and anabolic factors naturally involved in tissue repairs, PRP seems to be a reliable local treatment for musculoskeletal injuries such as tendon or cartilage lesions [5,6,7]. These tissues are differentiated from the others by their low healing abilities. Indeed, they have a very tenuous vascularization and cell density, restricting their repair capabilities. Damage of such tissues often leads to a slow and uncertain healing process. Moreover, numerous high-level studies demonstrated PRP efficacy and tolerance compared with hyaluronic acid injections or intra-articular corticosteroids, notably in knee osteoarthritis [8,9,10,11,12].

This therapy currently suffers from a substantial methodologic heterogeneity in the scientific literature. We also observe a lack of standardization in PRP preparation protocols and a fluctuant terminology. These variations lead to very different final injected products and clinical results [6,8,13,14,15,16]. This observation could be explained by incomplete knowledge concerning biologic properties of PRP and its action process [17,18].

In spite of the numerous studies existing in the literature, none specifically focused on the patient’s experience. This is why we wanted to provide an insight on this point: are patients undergoing PRP concretely satisfied with this treatment or not?

Our study aimed to evaluate patient satisfaction after a PRP injection for cartilage lesions of the knee in real-life conditions thanks to a specific score named SATMED-Q©.

The secondary endpoints consisted of the description of the population who received this product as well as the cellular composition of the final product that was administered. To put the satisfaction study into perspective with clinical outcomes, patients’ pain and function were also assessed.

## 2. Materials and Methods

The protocol of this study was validated by the Research and Innovation Department of the French Military Health Service and the Hospital Clinical Research Program unit. It was registered under the following number: 54-2020 HIA-CS. Our protocol completed the reference method MR004 with a declaration made at the CNIL (Commission Nationale de l’Informatique et des Libertés), the French national data protection agency.

Sas-PRP is a retrospective routine care study. It is an observational and evaluative work that was retrospectively conducted in the Physical and Rehabilitation Service of the French Instruction Military Hospital Percy of Clamart, Ile-de-France. We included all patients presenting a cartilage lesion of the knee who received at least one PRP injection for 16 consecutive months, between November 2019 and February 2021. “Cartilage lesions” was here used as a generic term to refer to knee osteoarthritis and focal cartilage lesions (focal chondropathy). All participants were affiliated with the French social security system. Patients who refused to participate and those who could not read or understand French were not included in the study.

### 2.1. Presentation of the Equipment

The sampling kit used for PRP preparation was the Hy-Tissue PRP 50^®^. This sampling kit is commercialized by Fidia Farmaceutici s.p.a. Both the sampling and injection kits were paid for by the patient. The Fidia society also provided the cellular centrifugation system named DUOGRAFTER II^®^ and the automatic blood analysis system Bio-QControl^®^. The blood analysis system was used for collecting cellular data on both whole blood samples and platelet-rich plasma. No option was added to the initial system.

### 2.2. Preparation and Injection Procedures (Table 1)

Peripheral blood was first collected in a 50 mL syringe containing 5 mL of a 3.8% sodium citrate solution. During the blood sample, the syringe was softly shaken every 5 mL in order to homogenize the anticoagulation. The blood sample ended when the 50 mL syringe was completed. The citrated blood was transferred into a tube of a similar volume to be centrifuged. The centrifugation lasted 8 min at 1800 rpm creating a 360 G centrifuge force. Only one centrifugation cycle was achieved. The platelet-poor supernatant was then removed from the tube. The PRP final volume was adjusted according to the whole blood platelet concentration. A cellular analysis was realized with three drops of PRP. The aim was to provide between 3 and 4 billion platelets to the knee. This dose was collectively determined, taking into account the manufacturer’s instructions as well as the compromise of achieving a high platelet dose within a limited volume. No activation factor was added to the PRP. The injection was immediately performed in an aseptic condition.

**Table 1:** Data related to the PRP preparation equipment.

**Volume of Blood Collected**	45 mL
**Nature and volume of the anticoagulant solution**	5 mL of sodium citrate solution 3.8%
**Centrifuge**	DUOGRAFTER II©
**Modification brought to the constructor’s protocol**	None
**Centrifugation speed**	1800 rpm
**Centrifugal speed in gravitational (g) force**	360 G
**Centrifugation time**	8 min
**Number of spin cycles**	1 cycle
**Blood analysis system (whole blood and PRP)**	Bio-QControl^®^ system; 3 whole blood and PRP drops are used for the analysis
**Platelet activation**	None
**Delay between sample and injection**	Immediate
**Infiltration condition**	Intra-articular injection without ultrasound guidance in aseptic conditions

### 2.3. Observation and Safety Precautions

To preserve platelets and their activity, patients were informed to not use NSAIDs (non-steroidal anti-inflammatory drugs) or oral corticosteroids 7 days before and after the procedure. Intra-articular corticosteroid injections should not have been performed for the last 6 weeks. It was not asked to stop an antiplatelet treatment.

No premedication or local analgesia was given before or during the infiltration. We could use MEOPA (Nitrogen Monoxide–Oxygen Mixture) that was delivered by a facial mask during the procedure according to the patient’s pain.

All patients were put under observation for 15 min after the injection before leaving.

### 2.4. Satisfaction Questionnaire and Algo-Functional Scores

Evaluation of patients’ satisfaction was performed with the Scaling and Scoring of the Treatment Satisfaction with Medicines Questionnaire (SATMED-Q©) [19]. This questionnaire has been scientifically validated in French [20]. It allowed us to explore six dimensions of a given treatment: adverse events, perceived treatment effectiveness, the convenience of use, its impact on daily activities, medical care, and global satisfaction. SATMED-Q© is composed of 17 items on a 5-point Likert-type scale from 0 to 4. The maximum score is 68. The higher the score, the better the patient’s satisfaction. We decided to put it on a 0 to 100 scale for a better comprehension and representation of results. Alongside, a direct rating of the treatment satisfaction was performed on a numerical scale from 0 to 10. Each patient was asked to declare the onset action of PRP and its length of effectiveness.

In addition to this satisfaction survey, we combined two adapted pain and function scores: the Western Ontario and McMaster University index (WOMAC) and the subjective questionnaire of the International Knee Documentation Committee (IKDC) [21,22].

We finally used the OMERACT-OARSI criteria [23] to identify patients who could be considered as treatment responders. The criteria were defined as:

1. Improvement in pain or in function ≥ 50% and absolute change ≥ 20;
2. Or improvement in at least two of the following:
  - Improvement in pain VAS score ≥ 20% and absolute change ≥ 10;
  - Improvement in function ≥ 20% and absolute change ≥ 10;
  - Improvement in patient’s global assessment ≥ 20% and absolute change ≥ 10.

The function assessment was performed by the WOMAC total score.

These different questionnaires were part of the current care proposed in our service. They are submitted before and between the sixth and twelfth month following a PRP infiltration.

### 2.5. Statistical Analysis

Discrete variables are described as numbers and percentages. Quantitative variables are presented as mean ± standard deviation (SD) or median and interquartiles (Q1;Q3).

Concerning quantitative variables, comparisons between groups were performed using the Wilcoxon Mann–Whitney and Student’s *t* tests. For ordinal variables, the Kruskal–Wallis test was used.

For the pain and function sub-group analysis, we decided not to perform statistical analysis if the results did not reach the MCII (minimal clinically important improvement) threshold or if the population was too tiny (≤5 subjects).

All tests were two-sided and a *p*-value < 0.05 was considered statistically significant.

## 3. Results

### 3.1. Descriptive Analysis

Between November 2019 and February 2021, 81 patients received at least one PRP intra-articular injection of their knee. The population is described in Table 2. Among the 81 patients included, two received a double injection separated for 10 days. All the others underwent a single-injection protocol. During the study, a total of 12 patients (14.8%) received more than one injection before M12. PRP characterization and doses are summarized in Table 3.

### 3.2. Platelet-Rich Plasma Treatment

According to the DEPA classification [15], the injected PRP was classified as BBA non-activated leukocyte-poor PRP (LP-PRP). The production process led to a very pure PRP containing > 95% platelets. This percentage corresponded to an average of 3.86 billion platelets (Table 3).

### 3.3. Follow-Up Survey

A total of 58 questionnaire replies were collected between M6 and M12. The mean response delay was 8.7 months (min: 6 months; max: 12 months).

#### 3.3.1. SATMED-Q Score

The mean SATMED-Q score was 80.8/100. This result appeared similar to the one obtained by direct rating which was 7.89/10. Scores of the different SATMED-Q dimensions are delivered in Table 4. Only two patients reported adverse events due to the PRP injection on the SATMED-Q. These side effects were described as mild-to-moderate.

The sub-groups analysis (Table 5) did not find any significant difference in terms of satisfaction according to gender, age, other PRP delivery before/after the one studied, or previous knee traumatic injury. However, we observed a significant difference depending on the delay since the complaint onset. Study subjects with older symptoms expressed a higher satisfaction after their PRP treatment.

#### 3.3.2. Pain and Function Scores

Results concerning patients’ pain and function showed an improvement in every score. None reached a significant difference (Table 6).

The OMERACT-OARSI criteria were analyzed for 37 patients thanks to the collected data. A total of 19 of them were considered as responders according to the pre-defined criteria.

The sub-group analysis of pain and function scores revealed significant differences between baseline and the last response (M6 to M12) in the population with focal cartilage lesions (chondropathy) and particularly those ≥40 years old (Table 7).

#### 3.3.3. Onset of Action and New Injection Delay

Patients declared a mean onset of action of 6.02 weeks (min: 0.29 weeks; max: 25 weeks) and deemed it appropriate.

Later monitoring data were available for 67 patients. A total of 28 of them were re-injected with PRP. The mean re-injection delay was 18.6 months ± 7.8 (min = 5 months; max = 36 months). Four patients were operated on: three total knee prostheses and one tibial valgus osteotomy.

## 4. Discussion

Our study population was relatively young compared to recent papers [10,11,24]. We found that it included a majority of men contrary to most other works dealing with platelet-rich plasma [9,10,11,24,25]. This could be explained by the large number of members of the armed forces we currently manage in our service. The significant musculoskeletal load due to their professional activities and their increased exposure to traumatic episodes are prone to lead to early and disabling lesions. This work also reflected the different indications of PRP injections on knee lesions. These included focal chondropathy in young patients and global osteoarthritis present in older people. This variety of patients illustrated our daily activity and probably that of many others all around the world. According to our experience, PRP was not used as a first-line therapy. The majority of patients had already undergone physiotherapy or other types of injections with HA or CSI. This may also be suggested by the small part of patients treated less than one year after their symptoms’ onset. To our knowledge, no high-quality scientific work has made recommendations regarding the best chronological order between these various treatment options [26]. Some authors found better efficiency while using HA and PRP together [27,28]. These results could be explained by better inflammation downregulation, particularly on the infrapatellar fat pad and the synovial membrane [29]. Nevertheless, other meta-analyses did not prove any superiority of PRP combined with HA compared to PRP alone [30]. Cellular analyses demonstrated a twofold increase in platelet concentration. The platelet increase factor largely depends on the centrifuge system [14,31,32]. Our increase factor was in the range exposed in the literature and is comparable to the ones obtained with the same centrifuge system [33,34,35]. Our PRP was classified as BBA according to the DEPA classification [15]. Even if we obtained a high dose of injected platelets (between 3 and 5 billion), we could have more platelets with higher PRP volumes and obtain >5 billion injected platelets (rank A). The production efficiency was 85% (rank B) and strictly depends on the device. The same production efficiency was declared by Silvestre et al. [34] who used the same device. We obtained a better PRP classification than Guillibert et al. who used the HighTissue PRP 20 device [33]. Our injection protocol was almost only a unique injection. This was an intended choice decided in line with the high PRP volume dispensed thanks to the PRP Hy-Tissue 50 kit. Currently, there are contradictory results with repeated PRP injections even in meta-analyses. For instance, McLarnon et al. [12] showed a better efficiency of PRP after a three-injection protocol realized in three consecutive weeks. Conversely, Hohman et al. [11] revealed a high protocol heterogeneity in the scientific literature without finding a better one. The mean re-injection delay was 18.6 months in our study and appeared suitable for patients. Moreover, thanks to a later look at our service database, it could concern more than 80% of our study population. The SATMED-Q score was very close to the one directly declared by patients on a numerical scale. This observation reflects the high pertinence of this score in the evaluation of treatment satisfaction on real-life monitoring. On the other hand, it also shows the reliability of a direct evaluation on a numerical scale that could be used in a daily activity as well. These results demonstrated very good satisfaction 6 to 12 months after a PRP delivery. Most of our patients seemed to benefit from PRP even after a year. We noticed through the different questionnaires that platelet-rich plasma acts on every aspect of the disability due to an osteochondral knee lesion: physical deficiency, action limitation, and participation restriction. Such conclusions were also made in other studies. MacLarnon and Heron [12] found significant improvements in pain and function with a reduction of 9.51 points in the WOMAC score and a 0.97-point decrease in the VAS score at M6. Identically, Chopin et al. [24] showed significant improvement at M7 in WOMAC and VAS scores with a minimal clinically important improvement (MCII) for VAS pain score achieved in 46.5% of patients at M7. We also observed the almost total absence of adverse events due to PRP in our study. Thus, the benefit–risk ratio appears to be in favor of its use in such indications [36]. Study subjects with older symptoms seemed to accrue more benefits from their infiltrations than others. This can illustrate a lower functional level at baseline in this particular population which can more appreciate the PRP action in daily activities. This inverse relationship between functional requirement and treatment satisfaction was also found according to age. We observed a better global satisfaction in the elderly population, without any significant difference, though. This gap could be understood as a higher functional strain in the young population both in professional and recreational activities. The delay between the injection and the perceived onset of action suggests questioning PRP efficiency after a minimal period of 6 weeks at risk of re-injecting patients too soon or leading to the erroneous conclusion of treatment inefficiency. This observation also invites us to question protocols with further infiltrations during this period. The global positive opinion expressed by our study population through SATMED-Q was logically accompanied by an improvement in all pain and function scores: VAS pain score, IKDC, and WOMAC. The mean improvement of these scores was similar to the ones in the literature [10,11,24,36]. The improvement in the WOMAC score was even clinically significant with an improvement of 11.89/100 points compared to the threshold of 9.11/100 points [37]. After treatment, the WOMAC score reached the patient-acceptable symptom state (PASS) contrary to baseline [38]. The VAS pain score was at the threshold of the MCII with an improvement of 1.76 points (vs. 1.99 points). Similarly, the VAS pain score was very close to the PASS threshold: 3.28 vs. 3.23/10 points. The sub-group analysis showed a greater pain and function improvement in the focal chondropathy compared to the osteoarthritis population. It converged towards two types of patients that seemed to particularly benefit from PRP. People with lower osteoarthritis damages (i.e., Kellgren–Lawrence grades 1–2) and older study subjects (i.e., >40 years old) with focal chondropathy were the patients who benefited most from their platelet-rich plasma injection. In the chondropathy population and particularly in patients > 40 years old, all pain and function scores were significantly improved. These results need to be put into the perspective of the tiny population composing the different sub-groups and by the repetitions of analyses. However, these findings are consistent with previous studies [34,39]. A total of 52% of the patients for whom we could gather the OMERACT-OARSI criteria were considered responders. This result is similar to the one obtained by Chopin et al. [24] which was 46.5% at M7 and by Silvestre et al. [34] which was 49%. Our study was limited by its low level of evidence. It probably includes multiple biases due to the absence of care standardization before and after the PRP injection as well as possible reporting errors and recall bias. Moreover, the respondents might have more benefits than the others. Despite that we found later data concerning most of our study population, we cannot discuss the future for the other patients who could have changed their physician or hospital and even underwent surgery. This study also did not take into account some predictors of response to PRP such as the body mass index (BMI), axial deformation of lower limbs, or heel-to-buttock distance [24,40]. These factors could have been analyzed in a sub-group analysis to identify patients who are most likely to benefit from platelet-rich plasma. We decided to use a satisfaction questionnaire due to the amount of pain and function scores used to assess PRP efficiency even on the same indications. This myriad of scores impairs the external consistency of studies while causing a lack of concordance in the results. Moreover, the single use of these scores exposes the risk of a questionable clinical relevance and could be a mismatch with the patient experience. Our study tends to illustrate the relevance of a satisfaction questionnaire in the daily clinical activity. The combined use of a satisfaction questionnaire and pain/function scores seems to be particularly interesting. To our knowledge, this is the first study focusing on patients’ satisfaction after a platelet-rich plasma injection in a cartilage lesion of the knee. This work’s value also consists in presenting an everyday population. According to our opinion, real-life studies are especially important if we want to improve the relevance of research in this field. It also highlights that it is possible to follow the experts’ recommendations concerning the follow-up, the preparation and the characterization of PRP apart from high-level studies. We could describe all the equipment used as well as our preparation protocol and the cellular data from total blood and final PRP were produced as recommended [8,14,16]. Thus, we also noted the good quality of the PRP obtained according to expert criteria [15].

## 5. Conclusions

Patients’ satisfaction after a PRP injection for a cartilage lesion of the knee seemed to be especially good in our real-life study with a mean SATMED-Q score of 80.8/100. Meanwhile, we observed a global improvement in pain and function scores notably in patients with early osteoarthritis lesions or older ones with focal chondropathy. In the global population, platelet-rich plasma appears to be both efficient and well tolerated. All in all, this product turns out to be a highly convincing therapeutic option for the management of knee lesions. Even if randomized controlled trials are the scientific studies’ gold standard, routine care studies also seemed especially relevant to assess this kind of therapeutic. Future research may lie in establishing databases or wide registers.

## Figures and Tables

**Table 2:** Description of the study population.

Population	n (%) Mean ± SD
**Gender**	
Male	50 (63%)
Female	31 (37%)
**Age**	48.3 ± 11.6 years (min: 20.3 y.o; max: 79 y.o)
**Time since symptom onset**	
Unspecified	22%
<1 year	11%
≥1 year and <3 years	25%
≥3 years et <5 years	14%
≥5 years	28%
**Pain localization**	
Single compartment	45%
Bicompartmental	14%
Global or unspecified	41%
**Other complaints**	
Joint swelling	34%
Instability	20%
Articular lock feeling	17%
**Diagnosis**	
Reported on consultation letter	41 (50%)
Medical imaging review	40 (50%)
***Focal chondropathy***	33
Internal femorotibial	17
Patellofemoral	16
External femorotibial	9
***Osteoarthritis (Kellgren-Lawrence)***	27
Grade 1	4
Grade 2	8
Grade 3	10
Grade 4	5
Combined meniscus injury	16
Isolated meniscus injury	5
Focal osteochondritis	2
**Previous treatment before PRP injection**	
Unknown	25%
Hyaluronic Acid (HA)/corticosteroids injection (CSI)	45%
Physiotherapy	11%
Surgery (knee arthroscopy, meniscectomy, valgus tibial osteotomy)	9%
None (PRP as a first line therapy)	23%

y.o, years old.

**Table 3:** Characteristics of injected platelet-rich plasma.

	Mean ± SD/n (%)
**Whole blood sample cellular concentrations**	
Red blood cells (10^9^/mL)	4.13 ± 0.54
Leukocytes (10^6^/mL)	5.20 ± 1.35
Platelets (10^6^/mL)	259.99 ± 57.00
**PRP characteristics**	
Volume (mL)	9.3 ± 1.8
	(min = 5; max = 15)
Platelet increase factor	1.70 ± 0.34
Red blood cells (10^9^/mL)	0.03 ± 0.13
Leukocytes (10^6^/mL)	0.80 ± 0.97
Platelets (10^6^/mL)	436.35 ± 114.84
Quantity of injected red blood cells (10^9^)	0.26 ± 1.0
Quantity of injected leukocytes (10^6^)	6.99 ± 7.95
Quantity of injected platelets (10^6^)	3861.87 ± 452.69
Red blood cells (%)	4.6 ± 0.1
Leukocytes (%)	0.2 ± 0.0
Platelets (%)	95.2 ± 2.1
**Adverse event**	
1 benign vasovagal episode	

**Table 4:** SATMED-Q scores.

Scores	From M6 to M12 Mean ± SD
**SATMED-Q (/100)**	**80.81 ± 16.00**
Undesirable side effects/12	11.67 ± 0.10
Treatment effectiveness/12	8.64 ± 0.31
Convenience of use/12	9.48 ± 0.06
Impact on daily activities/12	8.41 ± 0.60
Medical care/8	6.98 ± 0.19
Global satisfaction/12	9.80 ± 0.15
**Direct rating (/10)**	**7.89 ± 2.12**

**Table 5:** Sub-group analysis of the SATMED-Q score.

	Median [Q1;Q3] SATMED-Q Score
**Gender**	*p* = 0.40 *
Male	87 [73;93]
Female	78.5 [62.25;96]
**Age**	*p* = 0.10 °
<30 years old	87.5 [78;93.25]
≥30 and <40 years old	80 [66.75;81.75]
≥40 years old	87 [66;96]
**Time since symptom onset**	*p* = 0.025 °
<1 year	66 [66;88]
≥1 year and <3 years	76 [57;84]
≥3 years and <5 years	96 [83;97]
≥5 years	88 [76.25;95.50]
**Total injections received**	*p* = 0.46 *
1 injection	81 [62.5;94.5]
≥2 injections	88 [78.75;93.75]

*: Mann–Witney Wilcoxon tests. °: Kruskal–Wallis test.

**Table 6:** Variations over baseline in pain and function scores.

	M0 Mean ± SD	After PRP Injection (From M6 to M12) Mean ± SD
		*p* = 0.09 *
**VAS pain (/10)**	5.04 ± 2.03	3.28 ± 2.20
		*p* = 0.13 *
**WOMAC (/96)**	32.68 ± 17.71	22.73 ± 15.94
		*p* = 0.12 *
**IKDC (/100)**	46.27 ± 16.75	57.72 ± 17.10

* Student’s *t* test.

**Table 7:** Sub-group analysis of pain and function scores.

Nature of Lesions	M0 Mean/(n)	After PRP Injection (From M6 to M12) Mean/(n)	*p*-Value
**VAS pain (/10)**			
Osteoarthritis	5.03 (20)	4.42 (19)	*p* = 0.36
KL1–KL2	6.4 (5)	4.5 (4)	NR
KL3–KL4	4.77 (11)	4.6 (10)	NR
Focal chondropathy	4.98 (28)	3.58 (24)	*p =* 0.02 *
<40 y.o.	5.14 (7)	4.0 (6)	NR
≥40 y.o.	4.93 (21)	3.44 (18)	*p* = 0.003 *
**WOMAC score (/96)**			
Osteoarthritis	36.40 (20)	28.84 (19)	*p* = 0.33
KL1–KL2	28.4 (8)	24.25 (4)	NR
KL3–KL4	40.55 (10)	30 (10)	NR
Focal chondropathy	30.2 (30)	18.92 (24)	*p* = 0.01 *
<40 y.o.	35.12 (8)	23.33 (6)	*p =* 0.63
≥40 y.o	28.41 (22)	17.44 (18)	*p =* 0.02 *
**IKDC score (/100)**			
Osteoarthritis	45 (20)	45.67 (19)	NR
KL1–KL2	38.85 (5)	48.85 (4)	NR
KL3–KL4	45.25 (11)	44.94 (10)	NR
Focal chondropathy	46.44 (30)	61.59 (24)	*p* = 0.001 *
<40 y.o.	41.67 (8)	59.77 (6)	*p* = 0.42
≥40 y.o	48.17 (22)	62.2 (18)	*p* = 0.003 *

Student’s *t* test. * p value < 0.05; NR, non-realized; y.o., years old, KL, Kellgren–Lawrence.

## Data Availability

Not applicable.
